# Supplementary material for: The genome sequence of the Lesser weever, Echiichthys vipera (Cuvier, 1829) (Perciformes: Trachinidae)
Source: Wellcome Open Res. 2026 Apr 21;11:231. [Version 1] doi: 10.12688/wellcomeopenres.26380.1 (PMC13639354; doi:10.12688/wellcomeopenres.26380.1)
Supplement: Peer review history

# Reviewer report: Reviewer response for version 1

**DOI:** 10.21956/wellcomeopenres.29053.r153978

**Published:** 2026-10-05

**Authors:** Jayan Duminda M Senevirathna

**Affiliations:** 1 Uva Wellassa University, Badulla, Uva Province, Sri Lanka

The authors have successfully generated a genome assembly for the lesser weever, *Echiichthys vipera*, achieving approximately 98% BUSCO completeness. The genome has been assembled to the scaffold level, with 25 pseudochromosomal structures identified, including the X and Y sex chromosomes. The identification of the sex chromosomes represents an important contribution to the genomic resources available for this species.

 The resulting genome assembly provides a substantial and valuable resource for future studies on the biology, evolution, population genetics, sex chromosome evolution, and conservation of *E. vipera*. Overall, the high BUSCO completeness and identification of the sex chromosomes demonstrate the value of this genomic resource for future research on the species.

Are sufficient details of methods and materials provided to allow replication by others?

Yes

Is the rationale for creating the dataset(s) clearly described?

Yes

Are the datasets clearly presented in a useable and accessible format?

Yes

Are the protocols appropriate and is the work technically sound?

Yes

Reviewer Expertise:

Aquatic Genetics and Biotechnology

I confirm that I have read this submission and believe that I have an appropriate level of expertise to confirm that it is of an acceptable scientific standard.

# Reviewer report: Reviewer response for version 1

**DOI:** 10.21956/wellcomeopenres.29053.r154426

**Published:** 2026-06-18

**Authors:** Karim Gharbi

**Affiliations:** 1 Earlham Institute, Norwich, England, UK

This data note describes a chromosome-level genome sequence for the Lesser weever ( *Echiichthys vipera*) generated as part of the Darwin Tree of Life Project. The note includes a general overview of the species, including taxonomy, distribution, and relevance to venom research. The introduction is informative and the methods are detailed and comprehensive. Assembly statistics and assessment are consistent with those of a reference-grade genome assembled to chromosome-level pseudo-molecules. RNA-seq data was generated and deposited to ENA, but not used for annotation. Overall, this study represents an important contribution to fish and biodiversity genomics.

 The report is well written and edited, with only a few minor areas to comment on:

 - how to navigate to the specific DNA barcoding protocol (including primer sequences) that was used for the focal sample is not immediately obvious from the link provided

 - a note should be added to Table 3 to highlight that Z/W as a possible alternative to X/Y designations

 - chromosomes is repeated twice in the third sentence of the second paragraph in Assembly statistics

 - the number of scaffolds is quoted as both 132 (text) and 133 (snail plot), this may a typo or linked to the sex chromosomes are accounted for

Are sufficient details of methods and materials provided to allow replication by others?

Yes

Is the rationale for creating the dataset(s) clearly described?

Yes

Are the datasets clearly presented in a useable and accessible format?

Yes

Are the protocols appropriate and is the work technically sound?

Yes

Reviewer Expertise:

Genomics, bioinformatics

I confirm that I have read this submission and believe that I have an appropriate level of expertise to confirm that it is of an acceptable scientific standard.
